# Supplementary material for: Albuminuria Is Affected by Urinary Tract Infection: A Comparison between Biochemical Quantitative Method and Automatic Urine Chemistry Analyzer UC-3500
Source: Diagnostics (Basel). 2023 Nov 2;13(21):3366. doi: 10.3390/diagnostics13213366 (PMC10650489; doi:10.3390/diagnostics13213366)
Supplement: Supplementary file 1 [file diagnostics-13-03366-s001.zip › diagnostics-2609668-supplementary.pdf]

Supplement Table S1. Results of UTISA questionnaire after antibiotic treatment between different albuminuria groups.

Median (IQR)	Negative albuminuria (N=19)	Transient albuminuria (N=24)	Persistent albuminuria (N=11)	p-value
Q1 Frequency	0 (0–1)	0 (0–1)	0 (0–1)	0.682
Q2 Urgency	0 (0–1)	0 (0–1)	0 (0–1)	0.830
Q3 Dysuria	0 (0–1)	0 (0–0)	0 (0–0.75)	0.306
Q4 Incomplete emptying	0 (0–1)	0 (0–0)	0 (0–1)	0.143
Q5 Lower abdominal pain	0 (0–0)	0 (0–0)	0 (0–1)	0.410
Q6 Low back pain	0 (0–0)	0 (0–0)	0 (0–1)	0.292
Q7 Gross hematuria	0 (0–0)	0 (0–0)	0 (0–0)	0.398
Total score	1 (0–5.75)	1 (0–2.5)	1 (0.25–4)	0.486
